# Fibrinogen inhibits sonic hedgehog signaling and impairs neonatal cerebellar development after blood–brain barrier disruption

**DOI:** 10.1073/pnas.2323050121

**Published:** 2024-07-23

**Authors:** Olivia Weaver, Dawn Gano, Yungui Zhou, Hosung Kim, Reshmi Tognatta, Zhaoqi Yan, Jae Kyu Ryu, Caroline Brandt, Trisha Basu, Martin Grana, Belinda Cabriga, Maria del Pilar S. Alzamora, A. James Barkovich, Katerina Akassoglou, Mark A. Petersen

**Affiliations:** ^a^Department of Pediatrics, University of California San Francisco, San Francisco, CA 94158; ^b^Gladstone Institute of Neurological Disease, Gladstone Institutes, San Francisco, CA 94158; ^c^Center for Neurovascular Brain Immunology at Gladstone Institutes and University of California San Francisco, San Francisco, CA 94158; ^d^Department of Neurology, Weill Institute for Neurosciences, University of California San Francisco, San Francisco, CA 94158; ^e^Department of Neurology, Stevens Neuroimaging and Informatics Institute, Keck School of Medicine, University of Southern California, Los Angeles, CA 90033; ^f^Department of Radiology & Biomedical Imaging, University of California San Francisco, San Francisco, CA 94143

**Keywords:** neurovascular, coagulation, stem/progenitor cell, preterm Infant

## Abstract

Cerebellar injury in preterm infants with central nervous system (CNS) hemorrhage results in lasting neurological deficits and an increased risk of autism. The impact of blood-induced pathways on cerebellar development remains largely unknown, so no specific treatments have been developed to counteract the harmful effects of blood after neurovascular damage in preterm infants. Here, we show that fibrinogen, a blood-clotting protein, plays a central role in impairing neonatal cerebellar development. Longitudinal MRI of preterm infants revealed that cerebellar bleeds were the most critical factor associated with poor cerebellar growth. Using inflammatory and hemorrhagic mouse models of neonatal cerebellar injury, we found that fibrinogen increased innate immune activation and impeded neurogenesis in the developing cerebellum. Fibrinogen inhibited sonic hedgehog (SHH) signaling, the main mitogenic pathway in cerebellar granule neuron progenitors (CGNPs), and was sufficient to disrupt cerebellar growth. Genetic fibrinogen depletion attenuated neuroinflammation, promoted CGNP proliferation, and preserved normal cerebellar development after neurovascular damage. Our findings suggest that fibrinogen alters the balance of SHH signaling in the neurovascular niche and may serve as a therapeutic target to mitigate developmental brain injury after CNS hemorrhage.

## Significance

Central nervous system (CNS) hemorrhage, the most common brain injury in premature infants, significantly increases the risk of permanent neurological impairment. The developing cerebellum is particularly vulnerable to injury from CNS bleeds, for which no specific treatment exists. In this study, we found that fibrinogen, a clotting protein that crosses a damaged blood–brain barrier, inhibits sonic hedgehog signaling in cerebellar neuron progenitors to impede the formation of new neurons in the neonatal cerebellum. Remarkably, fibrinogen depletion attenuated neuroinflammation, enhanced neurogenesis, and improved cerebellar growth in models of preterm cerebellar injury. Thus, fibrinogen-targeted therapies may counteract the harmful effects of blood in the developing CNS and provide a specific treatment to improve neurodevelopmental outcomes in preterm infants with CNS hemorrhage.

Cerebellar injury is increasingly recognized as a major contributor to neurodevelopmental impairment in preterm infants (1–3). Although the focus historically has been on forebrain pathology, a growing body of evidence demonstrates that preterm infants with isolated cerebellar injury or impaired cerebellar growth, also known as cerebellar hypoplasia, have increased risk of cognitive, motor, language, and behavioral disabilities (2–6). The cerebellum undergoes a rapid phase of development during late gestation and into the postnatal period (7, 8). This growth is largely driven by sonic hedgehog (SHH)-induced proliferation of cerebellar granule neuron progenitors (CGNPs) (9–11). Disruption to cerebellar neurogenesis during this critical period can have profound and enduring effects on cerebellar function (12, 13). In fact, perinatal cerebellar injury is one of the strongest risk factors for the development of autism and attention deficits in preterm infants (1, 14, 15). Given the high rate of autism and attention deficit/hyperactivity disorder in children born preterm (16, 17), identification of factors that alter cerebellar development is necessary to develop new therapeutic strategies that prevent neurodevelopmental disabilities and improve behavioral outcomes in preterm infants.

Cerebellar hemorrhage (CBH) often occurs during this crucial period of cerebellar development in preterm infants (12, 18). CBH affects roughly 20% of extremely preterm infants, with the risk of severe bleeding increasing with earlier gestational age (GA) and lower birth weight (19, 20). Postnatal infections, such as sepsis and necrotizing enterocolitis (NEC), occur in around 20% of extremely preterm infants and also increase the risk of CBH (21, 22). CBH in preterm infants poses a high risk of adverse neurologic outcomes, even without other brain injuries (3). Moreover, the risk of abnormal outcomes rises as the size of CBH increases (2, 23). Although previous studies have shown a correlation between CBH and neurodevelopmental impairments (2–4), they have not addressed the fundamental question of how pathological changes in the neurovascular niche may be driving preterm cerebellar injury and if any component of blood can be targeted for therapeutic purposes to promote normal cerebellar development.

Fibrinogen, a blood coagulation protein, crosses a leaky blood–brain barrier (BBB) and is deposited in the perivascular extracellular matrix (ECM) in a wide range of neurological diseases (24). Fibrinogen has critical roles in the adult CNS in neuroinflammation (25–27), glial scar formation (28), promotion of neurodegeneration (29–33), and inhibition of neurorepair (34–37). Fibrinogen has direct inhibitory effects on CNS progenitor cells, activating signaling pathways that alter cell fate and inhibit myelin regeneration and cortical neurogenesis (34–36). When fibrinogen enters the CNS, it is also converted to insoluble fibrin, which induces oxidative stress and proinflammatory activation of microglia and macrophages (26, 27, 33), which contributes to neuronal damage and failure of CNS regeneration (24, 38). Despite the evidence for fibrinogen’s pathogenic effects in the adult CNS, the role of fibrinogen in the developing brain has not been explored.

Here, we show that fibrinogen is a critical mediator of preterm cerebellar injury after neurovascular damage. Human preterm infant MRI and mouse models of neonatal cerebellar injury revealed that BBB disruption and fibrinogen deposition create an inhibitory microenvironment that impedes cerebellar growth. We found that fibrinogen inhibited SHH signaling and proliferation of CGNPs to impair neonatal cerebellar neurogenesis, revealing a blood-induced pathway that may be therapeutically targeted to promote normal CNS development after neurovascular damage. Indeed, fibrinogen depletion enhanced CGNP neurogenesis and restored cerebellar growth after neonatal cerebellar injury. Thus, fibrinogen-targeted therapies may counteract the harmful effects of blood in the developing CNS and provide a potential therapeutic avenue to improve neurodevelopmental outcomes in preterm infants with neurovascular damage.

## Results

### CBH Disrupts Cerebellar Growth in Preterm Infants.

**Fig. 1.**
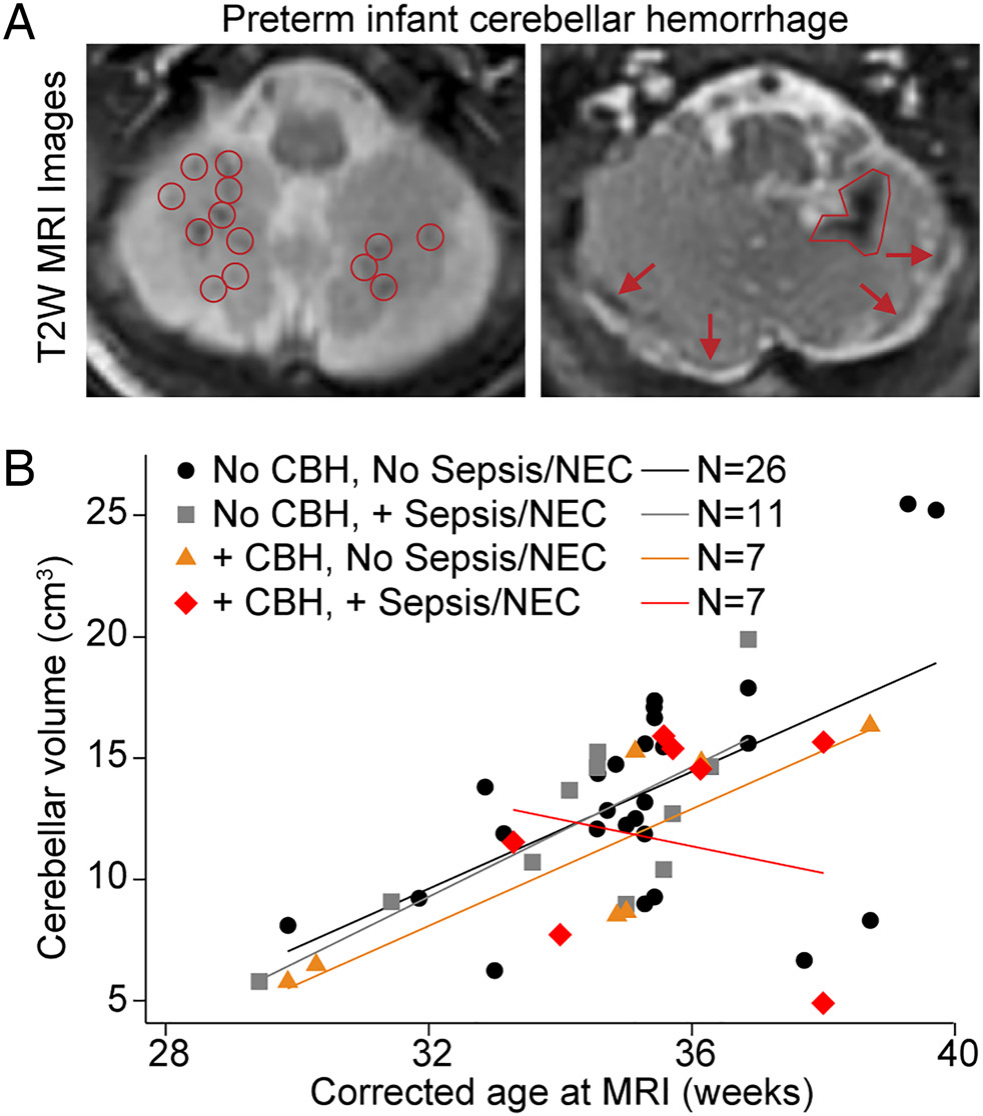
CBH disrupts cerebellar growth in preterm infants. (*A*) *Left,* T2-weighted MRI of cerebellum from an infant born at GA 24 wk + 2 d and imaged at corrected GA 39 wk + 5 d. Red circles, multiple small hemorrhages throughout the hypoplastic cerebellum. *Right*, T2-weighted MRI of cerebellum from an infant born at GA 24 wk + 5 d and imaged at corrected GA 42 wk. Red outline, large area of previous parenchymal hemorrhage associated with regional volume loss. Red arrows, linear areas of hemorrhage on surface of the hypoplastic cerebellum. (*B*) Scatter plot and mean fit lines of cerebellar volumes over the corrected GA. CBH, moderate/severe CBH; + sepsis, blood-culture positive sepsis; NEC, necrotizing enterocolitis.

We analyzed 59 extremely and very preterm infants (mean birth GA 28.59 ± 1.91 wk) imaged with MRI at a median of 35.28 wk (IQR 33.57 to 36.14), of whom 22 (37%) had CBH on MRI. CBH was mild in 7/22 (32%) and moderate/severe in 15 (68%), which included extensive multifocal hemorrhages, large parenchymal bleeds, and blood deposition on the cerebellar surface (Fig. 1*A*). Younger GA and lower birth weight were associated with increased rate of CBH (*SI Appendix*, Table S1), as previously reported (20). Postnatal infection, a significant risk factor for adverse neurodevelopmental outcomes in preterm infants (39), was associated with a twofold higher risk of CBH (risk ratio 1.9, 95% CI 0.97 to 3.6).

Cerebellar hypoplasia in preterm infants has been associated with long-term motor abnormalities, worse cognitive scores, delayed language development, and attention and socialization deficits (40). To assess the relationship of CBH, postnatal infection, and cerebellar volume near term-equivalent age, multivariate linear regression adjusting for known confounders of cerebellar volume was used (*SI Appendix*, Table S2). Moderate/severe CBH was independently associated with decreased cerebellar volume near term-equivalent age (beta: −1036.44 mm^3^, 95% CI −2077.89 to 5.0, *P* = 0.051), adjusting for GA, age at MRI, total cerebral volume, sepsis, and exposure to postnatal steroids. Interestingly, postnatal infection, defined as culture-positive sepsis and/or NEC, was not associated with decreased cerebellar volume unless CBH was present (Fig. 1*B*), suggesting BBB disruption and deposition of blood proteins in the CNS may be critical to the pathogenesis of cerebellar hypoplasia. Indeed, CBH alone was associated with lower cerebellar volumes, and the combination of CBH and postnatal infection had the greatest effect on cerebellar growth (Fig. 1*B*) with a more than 2-fold reduction in cerebellar volume (beta: −2310.3 mm^3^, 95% CI −4177.2 to −443.4, Interaction *P* = 0.016). The effect of CBH and postnatal infection on cerebellar volume remained significant even after excluding infants with intraventricular hemorrhage (IVH) (beta: −3833.6 mm^3^, 95% CI −6365.4 to −1301.8, Interaction *P* = 0.004), suggesting CBH inhibits cerebellar growth independent of cerebral IVH. These results implicate neurovascular pathology as a link between inflammatory and hemorrhagic injuries to the preterm cerebellum and support elucidating the role of blood proteins in preterm cerebellar hypoplasia.

### Fibrinogen Disrupts Cerebellar Growth In Vivo.

Cerebellar growth is driven by neurogenesis from CGNPs which cover the surface of the cerebellum to form the external granular layer (EGL) (11–13). In adult cortical injury models, fibrinogen enters the CNS across a leaky BBB and inhibits neurogenesis in the subventricular zone (36). We hypothesized that fibrinogen may also suppress neurogenesis in the developing cerebellar EGL after neurovascular disruption or hemorrhage and inhibit cerebellar growth.

**Fig. 2.**
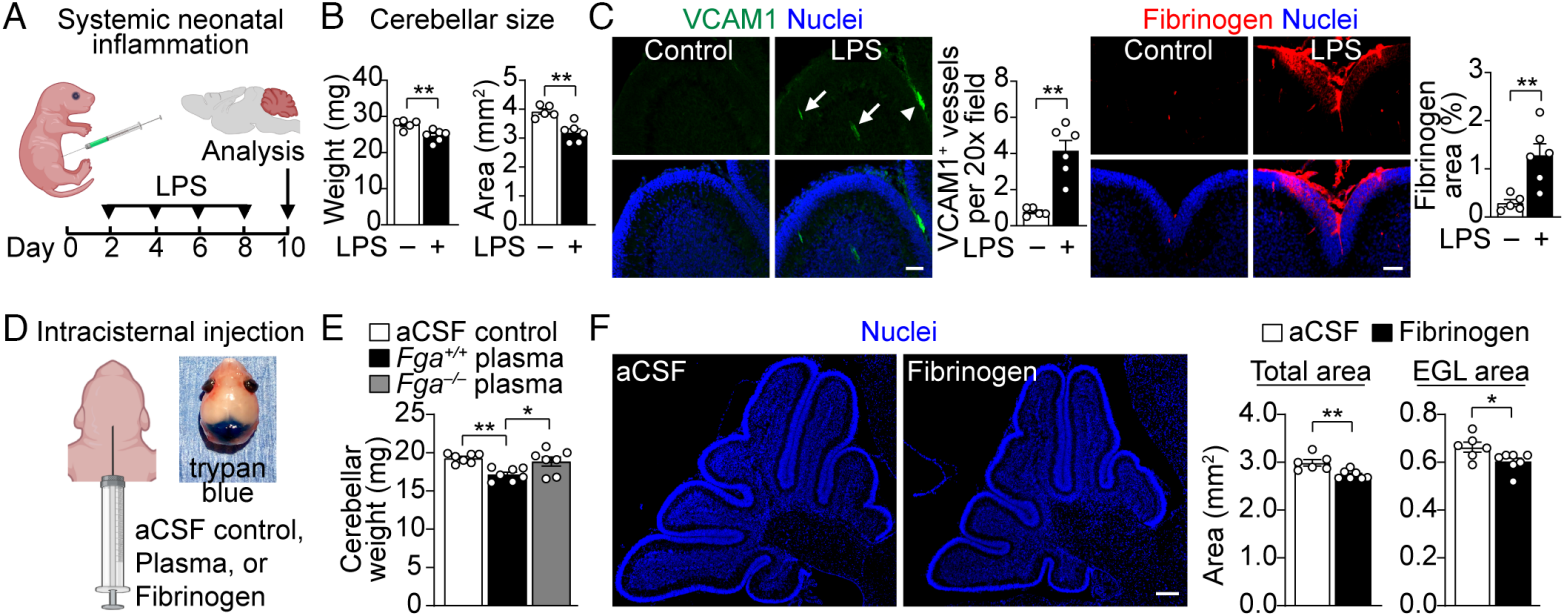
Fibrinogen is deposited in the cerebellum after systemic neonatal inflammation and disrupts cerebellar growth. (*A*) Schematic of LPS-induced neonatal inflammation. Created with BioRender.com. (*B*) Cerebellar weight and cross-sectional area at P10. Data are mean ± SEM from n = 5 to 6 mice per group. **P < 0.01, Mann–Whitney. (*C*) VCAM1^+^ (green) blood vessels in parenchyma (arrows) and meninges (arrowhead) and fibrinogen (red) in P10 cerebellum, nuclei (blue) labeled with DAPI. (Scale bar, 50 µm.) Data are mean ± SEM from n = 5 to 6 mice per group. **P < 0.01, Mann–Whitney. (*D*) Schematic of IC injection (2 µL) at P2 (*Left*) and visualization of trypan blue in posterior fossa (*Right*). aCSF, artificial cerebrospinal fluid. Created with BioRender.com. (*E*) Cerebellar weight at P7. *Fga*^+/+^, plasma from wild-type control mice. *Fga^−/−^*, plasma from fibrinogen knockout mice. Data are mean ± SEM from n = 7 mice per group. *P < 0.05, **P < 0.01, one-way ANOVA with Tukey. (*F*) P7 cerebellum, nuclei (blue) labeled with DAPI. aCSF or Fibrinogen (2 µL, 5 mg/mL) was injected at P2. (Scale bar, 200 µm.) Data of total cerebellar and EGL area are mean ± SEM from n = 6 (aCSF) and 7 (fibrinogen) mice per group. *P < 0.05, **P < 0.01, Mann–Whitney.

Rodent cerebellar development in the first two postnatal weeks closely mirrors that of the human between 24 and 40 wk gestation (13), providing a well-characterized developmental window to model preterm cerebellar injury. We first examined a mouse model of systemic neonatal inflammation in which bacterial lipopolysaccharide (LPS) is injected intraperitoneal (IP) every other day from postnatal day (P)2-P8 (Fig. 2*A*) to mimic repeated inflammatory insults, which has been linked to increased risk of CBH and poor neurologic outcomes in preterm infants (39). Consistent with prior work (41), LPS-injected mice had more cerebellar microglia/macrophages (*SI Appendix*, Fig. S1*A*) and significantly smaller cerebella at P10 (Fig. 2*B*), indicating increased neuroinflammation and impaired cerebellar growth, respectively. LPS increased vascular cell adhesion molecule 1 (VCAM1)^+^ blood vessels, a marker of neurovascular inflammation, and fibrinogen deposition, indicating BBB disruption, in the cerebellum, which was prominent in the EGL (Fig. 2*C*).

Given the fibrinogen deposition observed in the LPS model, we next conducted intracisternal (IC) injection studies to directly assess the impact of blood components on cerebellar development. As the rapidly proliferating CGNPs in the outer EGL are in contact with cerebrospinal fluid (CSF) (12, 42), we performed IC injections of plasma, fibrinogen, or artificial CSF (aCSF, negative control) in P2 mice to determine effects on the EGL and cerebellar growth (Fig. 2*D*). Plasma from wild-type mice (*Fga*^+/+^) significantly decreased cerebellar weight at P7, indicating impaired cerebellar growth (Fig. 2*E*). In contrast, fibrinogen-deficient (*Fga^−/−^*) plasma, which lacks fibrinogen, did not significantly reduce cerebellar size (Fig. 2*E*), suggesting that fibrinogen is a key protein in the blood that disrupts cerebellar growth. Indeed, IC injection of fibrinogen alone was sufficient to increase neuroinflammation at P3 (*SI Appendix*, Fig. S1*B*) and decrease total cerebellar and EGL area at P7 (Fig. 2*F*). These results suggest that fibrinogen extravasation in the neonatal CNS after inflammatory and hemorrhagic injuries inhibits cerebellar development.

### Fibrinogen Inhibits the SHH Pathway and Proliferation of CGNPs.

**Fig. 3.**
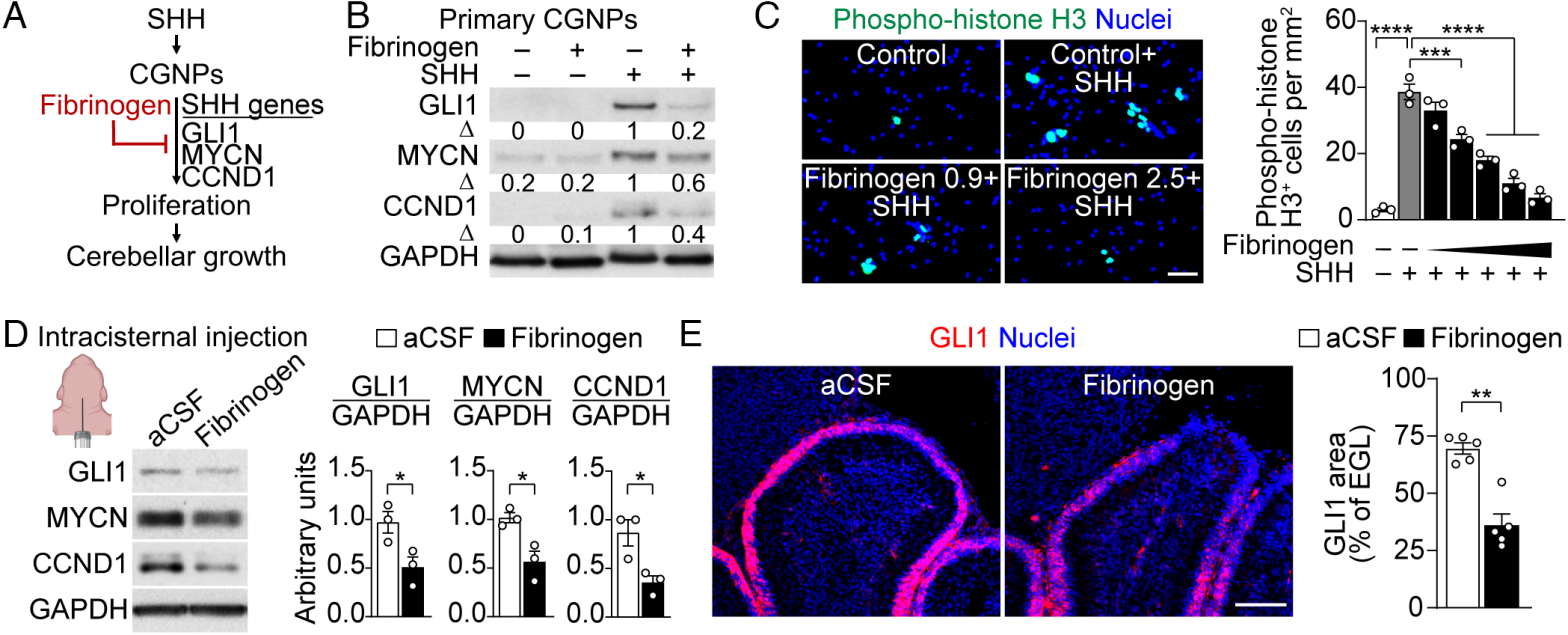
Fibrinogen inhibits the SHH pathway and proliferation of CGNPs. (*A*) Schematic of SHH effects in CGNPs and fibrinogen inhibition. (*B*) GLI1, MYCN, and CCND1 protein levels 24 h after control or SHH (3 µg/mL) treatment of primary CGNPs in the presence of fibrinogen (2.5 mg/mL). Blot representative of n = 2 independent experiments. Δ, densitometry fold change values. (*C*) Mitosis marker phospho-histone H3 (green) and nuclei (blue, DAPI) 48 h after control or SHH (3 µg/mL) treatment of primary CGNPs in the presence of fibrinogen (0.1, 0.3, 0.9, 1.5, 2.5 mg/mL). (Scale bar, 50 µm.) Data are mean ± SEM from n = 3 independent experiments. ***P < 0.001, ****P < 0.0001, one-way ANOVA with Dunnett. (*D*) GLI1, MYCN, and CCND1 protein levels in cerebellum 24 h after IC injection of aCSF or fibrinogen (2 µL, 5 mg/mL) at P2. Densitometry fold change values are mean ± SEM from n = 3 mice per condition. *P < 0.05, unpaired *t* test. Created with BioRender.com. (*E*) SHH activation marker GLI1 (red) and nuclei (blue, DAPI) in cerebellum 24 h after IC injection of aCSF or fibrinogen (2 µL, 5 mg/mL) at P2. (Scale bar, 100 µm.) Data are mean ± SEM from n = 5 mice per group. **P < 0.01, Mann–Whitney.

CGNP proliferation in the EGL is driven by SHH produced by Purkinje cells (9, 12). SHH induces the target genes *Gli1*, *Mycn*, and *Ccnd1* in CGNPs to promote cell cycle progression and cerebellar growth (9, 10) (Fig. 3*A*).

To determine fibrinogen’s effects on the SHH pathway in CGNPs, primary CGNPs were isolated from P5 to P7 mice, a period of peak proliferation in the EGL (43), and treated with SHH alone or with SHH plus fibrinogen (2.5 mg/mL, a physiologic blood concentration) (34, 35). Protein levels of GLI1, MYCN, and CCND1 at 24 h posttreatment were measured as markers of SHH pathway activation, and cell counts at 3 d posttreatment served as an indirect measure of CGNP proliferation. Fibrinogen significantly reduced SHH pathway activation and CGNP cell number with no evidence of cell death (Fig. 3*B* and *SI Appendix*, Fig. S2 *A* and *B*).

Building on these findings, we explored the dose-dependent effects of SHH and fibrinogen on CGNP proliferation, as measured by the mitosis marker phospho-histone H3. We determined that a SHH dose of 3 µg/mL exerted a maximal mitogenic effect on CGNPs (*SI Appendix*, Fig. S3*A*), consistent with prior studies (9, 10). In the presence of 3 µg/mL SHH, fibrinogen caused a dose-dependent inhibition of CGNP proliferation with an IC50 of 0.99 mg/mL and significant inhibition even at 0.3 mg/mL (Fig. 3*C*), a concentration eightfold lower than typical fibrinogen blood levels in preterm infants (44). Furthermore, when coated onto culture plates as insoluble fibrin, concentrations as low as 1 µg/mL markedly suppressed SHH-induced CGNP proliferation (*SI Appendix*, Fig. S3*B*), suggesting that even small amounts of fibrin(ogen) in the ECM can have potent inhibitory effects on SHH-induced CGNP proliferation. Importantly, a SHH dose of 6 µg/mL, which is double the maximal mitogenic concentration, did not rescue the inhibition caused by a half-maximal dose of fibrinogen (*SI Appendix*, Fig. S3*A*), demonstrating that high levels of SHH do not ameliorate fibrinogen’s inhibitory effect on CGNP proliferation.

Notably, IC injection of fibrinogen also suppressed the SHH pathway in the cerebellum in vivo with an approximately twofold decrease in GLI1, MYCN, and CCND1 24 h after injection (Fig. 3 *D* and *E*). These results support fibrinogen as the major component of blood that disrupts SHH signaling and inhibits CGNP neurogenesis in the cerebellum after BBB disruption.

### Fibrinogen Depletion Rescues Cerebellar Pathology in Systemic Neonatal Inflammation.

**Fig. 4.**
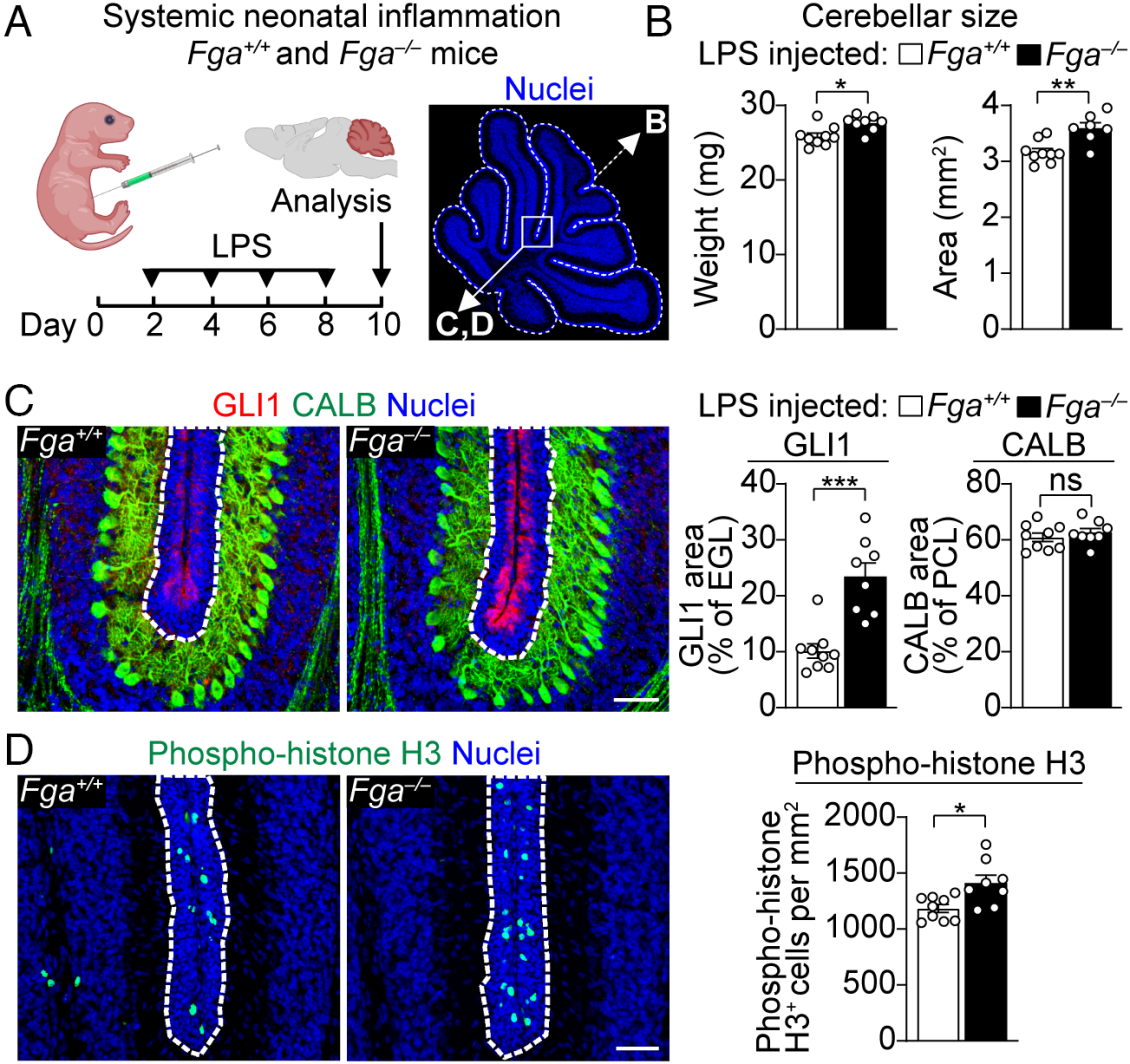
Fibrinogen depletion rescues cerebellar pathology in systemic neonatal inflammation. (*A*) Schematic of LPS-induced neonatal inflammation. Created with BioRender.com. Image of P10 cerebellum with nuclei (blue) labeled with DAPI to show representative cerebellar cross-sectional area (dotted white line) quantified in *B* and image areas (solid white square) shown in *C* and *D*. (*B–D*) *Fga*^+/+^, LPS-treated littermate controls. *Fga^−/−^*, LPS-treated fibrinogen knockout mice. Data are mean ± SEM from n = 9 (*Fga*^+/+^) and 7 to 8 (*Fga^−/−^*) mice per group. ns, not significant, *P < 0.05, **P < 0.01, ***P < 0.001, Mann–Whitney. (*B*) Cerebellar weight and cross-sectional area at P10. (*C*) SHH activation marker GLI1 (red), Purkinje cell marker CALB (green), and nuclei (blue, DAPI) in P10 cerebellum. Dotted white line outlines the EGL. (Scale bar, 50 µm.) (*D*) Proliferation marker phospho-histone H3 (green) and nuclei (blue, DAPI) in P10 cerebellum. Dotted white line outlines the EGL. (Scale bar, 50 µm.)

To determine the contribution of fibrinogen to cerebellar pathology in vivo, we analyzed fibrinogen knockout mice (*Fga^−/−^*) and littermate controls (*Fga*^+/+^) in the LPS neonatal inflammation model with BBB disruption (Fig. 4*A*). Remarkably, *Fga^−/−^* mice were protected from neuroinflammation (*SI Appendix*, Fig. S4) and had improved cerebellar growth (Fig. 4*B*) compared to *Fga*^+/+^ controls, suggesting a crucial role for fibrinogen in the pathogenesis of cerebellar hypoplasia. Accordingly, *Fga^−/−^* mice had increased SHH pathway activation in the EGL, as measured by the SHH target gene GLI1 (Fig. 4*C*), and more proliferating cells in the EGL, as measured by phospho-histone H3 (Fig. 4*D*). No differences were found in calbindin^+^ Purkinje cells (Fig. 4*C*), the major cellular source of cerebellar SHH (13), suggesting fibrinogen acts within the EGL to inhibit SHH signaling in CGNPs and suppress neurogenesis after BBB disruption.

## Discussion

CBH, a common brain injury in preterm infants, alters cerebellar development and significantly increases the risk of long-term neurological impairment (12, 18, 19). Currently, there are no specific treatments to counteract the harmful effects of blood in the developing brain. Our study revealed that the blood protein fibrinogen plays a significant role in inhibiting cerebellar neurogenesis and growth after neurovascular disruption. Fibrinogen promoted neuroinflammation, suppressed the SHH signaling pathway, and impeded the proliferation of CGNPs, ultimately leading to cerebellar hypoplasia. Importantly, genetic depletion of fibrinogen protected against cerebellar pathology and facilitated normal cerebellar development in neonatal mice after neurovascular damage. Moreover, our MRI analysis of preterm infants highlighted the significant link between CBH and cerebellar hypoplasia. Thus, targeting fibrinogen holds therapeutic promise for mitigating brain injury in preterm infants after hemorrhagic or inflammatory CNS injuries and has the potential to improve neurodevelopmental outcomes in this high-risk population.

Our study introduces fibrinogen as a key regulator of SHH signaling, the main driver of cerebellar neurogenesis (9, 45). Fibrinogen deposition in the developing CNS may inhibit SHH signaling through several mechanisms. As a component of the ECM, fibrinogen may control the bioavailability of SHH by blocking its interaction with other ECM proteins such as heparan sulfate proteoglycans (HSPGs) or laminin. These interactions are vital for the complete mitogenic effects of SHH, as they facilitate SHH localization to neurogenic niches and enhance its interaction with signaling complexes in the progenitor cell membrane (46–48). Like SHH, fibrinogen binds HSPGs through a heparin-binding domain (49, 50), so fibrinogen may compete with SHH for HSPG binding which would limit SHH bioavailability at the cell surface. Likewise, fibrinogen binds to beta1 integrins expressed in CGNPs (48, 51), so fibrinogen–integrin interactions could impair the recruitment of SHH–laminin complexes to the surface of CGNPs and limit their proliferative response (48). Fibrinogen may also activate receptor signaling pathways that inhibit intracellular SHH signaling cascades in CGNPs. Fibrinogen induces bone morphogenetic protein (BMP) receptor signaling in various CNS progenitor cells, including oligodendrocyte progenitor cells (OPCs) and neural progenitor cells (NPCs), to direct cell fate at sites of BBB disruption (34–36). In the developing cerebellum, BMP receptor activation opposes SHH signaling, in part, through activation of SMAD5 and downregulation of the transcription factors ATOH1/MATH1, GLI1, and MYCN, which control CGNP differentiation and proliferation (52–54). CNS hemorrhage activates BMP signaling in the preterm brain (55), raising the intriguing possibility that fibrinogen may orchestrate the balance of BMP and SHH signaling in the developing CNS at sites of neurovascular damage. Future studies will determine the mechanism by which fibrinogen inhibits SHH signaling in CNS progenitors.

Our study highlights several opportunities for further investigation. It is not known whether the cerebellar hypoplasia induced by fibrinogen persists in adulthood or is associated with long-term behavioral changes, as we only examined cerebellar pathology through the first ten postnatal days. In other models of cerebellar hypoplasia, such as chronic sublethal hypoxia, perturbing early postnatal cerebellar development leads to lasting changes in cerebellar size and function (56, 57), suggesting that injuries during this critical developmental window could result in lifelong cerebellar pathology and adverse behavioral outcomes. Moreover, we focused on cerebellar neurogenesis and injury given the well-characterized sequence of rodent postnatal cerebellar development (13), but it is likely that systemic inflammation and fibrinogen/plasma injection models could affect forebrain development and other CNS progenitors, such as OPCs and NPCs. Our study also does not rule out the possibility that *Fga^−/−^* mice are protected from cerebellar hypoplasia through pleiotropic mechanisms involving multiple pathways beyond the promotion of SHH signaling in CGNPs. For instance, brain myeloid cells can support neurogenesis and maturation of the developing CNS through secretion of growth factors such as IGF1 and sculpting of neuronal circuits (58, 59). After fibrinogen crosses the BBB, it is converted to fibrin, which exposes a cryptic epitope that binds and activates the CD11b/CD18 integrin receptor on microglia and macrophages (24). This activation results in the release of proinflammatory cytokines and reactive oxygen species (26, 27, 33), potentially tipping the balance of CNS innate immune populations away from their normal developmental roles and promoting an inhibitory microenvironment in areas of neurovascular damage. Indeed, fibrin-primed macrophages inhibit OPC differentiation to myelinating cells (34), suggesting a role for fibrin-induced inflammation in the inhibition of neurorepair. Further research is needed to determine the contribution of fibrin-induced neuroinflammation to altered cerebellar development after CBH.

Fibrinogen-targeted therapeutics may provide an avenue to counter the detrimental effects of blood in the CNS. Our findings indeed demonstrate that reducing fibrinogen protects cerebellar neurogenesis and growth after BBB disruption. However, the clinical use of fibrinogen-depleting agents or anticoagulants in a preterm population would pose a considerable bleeding risk, potentially overshadowing any neuroprotective benefits. Therefore, therapies focused on fibrinogen–receptor interactions and downstream signaling pathways may overcome the inhibitory neurovascular niche in preterm infant brain injury without compromising coagulation. Notably, fibrin-targeted immunotherapy has been shown to reduce innate immune activation and oxidative damage in adult neuroinflammatory and neurodegenerative models without anticoagulant side effects (27). Likewise, therapeutics that selectively block fibrinogen-induced BMP receptor signaling improve myelin repair and neurogenesis after CNS demyelinating or traumatic injuries without depleting fibrinogen (34–36). In light of our finding that fibrinogen impedes SHH signaling, it may be advantageous to combine these fibrinogen-targeted approaches with therapeutic SHH agonists, which promote BBB integrity (60, 61) and provide neuroprotection in models of neonatal brain injury (43, 56, 62), to maximize therapeutic benefit in patients with neurovascular damage or CNS hemorrhage. Future studies will unravel the intricate interplay between fibrinogen, SHH signaling, and progenitor cell dysfunction at the neurovascular interface, which may guide targeted therapeutic strategies to alleviate developmental brain injury after BBB disruption.

## Materials and Methods

### Human Subjects and MRI.

Study inclusion/exclusion criteria and methods of clinical data acquisition, MRI acquisition and analysis, cerebellar volume determination, and CBH classification are described in our prior studies (20, 63) and *SI Appendix*. The full study protocol, including the consent procedures, was approved by the UCSF Committee on Human Research, and parental consent was obtained accordingly.

### Mice.

C57BL/6 mice were obtained from The Jackson Laboratory. Fibrinogen-deficient (*Fga^−/−^*) mice (64) were obtained from Dr. Jay Degen (University of Cincinnati, OH). Adult mice were housed in groups of five per cage under standard vivarium conditions and a 12-h light/dark cycle. For breeding, a single male and female were paired to yield one litter per cage. For fibrinogen mutant mice, heterozygous (*Fga*^+/−^) mice were mated to obtain fibrinogen-deficient (*Fga^−/−^*) and littermate control (*Fga*^+/+^) mice for experiments. Male and female mouse pups of the same age and weight were randomized to treatment groups (*SI Appendix*, Table S3). No statistical methods were used to predetermine sample size, but sample sizes are similar to those reported previously. The exact number of animals per group is shown in each figure and legend. All data points were included for analysis. Injections, data collection, and quantification were performed by researchers blind to study group and animal genotypes. All animal protocols were approved by the UCSF Committee of Animal Research and in accordance with the NIH and ARRIVE guidelines.

### Systemic Neonatal Inflammation.

Systemic inflammation was induced with LPS (41, 65, 66). The day of birth was denoted as P0. Litters were culled to a maximum of eight pups and maintained in birth cage throughout the experiment. P2 male and female pups with similar weights were randomized into treatment groups (*SI Appendix*, Table S3). LPS (1 mg/kg, *Escherichia coli* O111:B4, Millipore Sigma) or sterile normal saline (vehicle control) was injected IP with a 32-gauge, 10-μL syringe (Hamilton) on P2, P4, P6, and P8. Mice were killed at P10 and brains were processed for immunofluorescence (IF) or immunoblotting. Cerebella were dissected and weighed prior to freezing.

### IC Injections in Neonatal Mice.

P2 C57BL/6 male and female pups with similar weights were randomized into treatment groups (*SI Appendix*, Table S3), cryoanesthesized, transilluminated to visualize brain structures, and 2 µL solutions injected into the cisterna magna with a 32-gauge, 10-μL syringe (Hamilton). Injections were aCSF (negative control), fibrinogen (5 mg/mL, #341578, Millipore-Sigma), plasma from *Fga*^+/+^ mice, or plasma from *Fga^−/−^* mice. Plasma was isolated as previously described (25, 26). All solutions contained 0.05% trypan blue dye to visualize successful injections. Pups were returned to their home cage, killed at P3 or P7, and cerebella processed for cerebellar weight, IF, or immunoblotting.

### Primary CGNP Cultures.

Mouse CGNPs were isolated as described (67) with modifications detailed in *SI Appendix*. Cells were treated with SHH (#464-SH, R&D Systems) alone or in the presence of fibrinogen (#341578, Millipore-Sigma) at concentrations indicated in the figure legends for up to 3 d in culture. Fibrin coating was prepared as described (26) with details in *SI Appendix*. Cells were processed for IF or immunoblotting as detailed in *SI Appendix*.

### IF and Immunoblots.

IF and immunoblot methods are described in *SI Appendix*. Primary antibodies for IF were CALB (1:200, rabbit monoclonal, #13176, Cell Signaling Technology), fibrinogen (1:1,000, rabbit polyclonal, gift from J. Degen, Cincinnati), GLI1 (1:100, goat polyclonal, #AF3455, R&D Systems), phospho-Histone H3 (1:250, rabbit monoclonal, #53348, Cell Signaling Technology), IBA1 (1:500, rabbit polyclonal, #019-19741 Wako), and VCAM1 (1:100, rat monoclonal, #550547, BD Pharmingen). Primary antibodies for immunoblots were CCND1 (1:1,000, rabbit monoclonal, #55506, Cell Signaling Technology), GAPDH (1:2,000, rabbit monoclonal, #2118, Cell Signaling Technology), GLI1 (1:1,000, mouse monoclonal, #2643, Cell Signaling Technology), and MYCN (1:1,000, rabbit monoclonal, #84406, Cell Signaling Technology).

### Statistical Analysis.

For MRI studies, a cross-sectional analysis was performed to evaluate the relationship between infection, severity of CBH, and cerebellar growth. Descriptive statistics were used to compare the rate of infection among those with CBH and those without. Multivariable linear regression modeling with variable selection defined a priori was used to evaluate the individual and joint effects of CBH severity and infection on cerebellar volume, adjusting for known confounders of cerebellar volume, including GA, postnatal age at MRI, exposure to postnatal steroids, and total brain volume. To evaluate individual independent associations, CBH severity and infection were included in the model with the confounding covariates. To evaluate the joint effects of CBH severity and infection on cerebellar volume, an interaction term was generated and included in a separate multivariable linear regression model including the prespecified confounding variables (68).

For animal studies, statistical analyses were performed with GraphPad Prism (Version 8). Data are presented as mean ± SEM. Statistical significance was determined with two-sided unpaired student’s *t* test, or two-sided Mann–Whitney test, or a one-way or two-way ANOVA followed by Dunnett’s or Tukey’s posttest for multiple comparisons as indicated in the figure legends. For in vitro studies, the number of independent experiments or biologic replicates is indicated in each figure and legend. A significance level of *P* ≤ 0.05 was used for animal and in vitro studies and *P* ≤ 0.10 for human MRI analyses.

## Supplementary Material

Appendix 01 (PDF)

## Data Availability

All study data are included in the article and/or *SI Appendix*.
