# Supplementary material for: Fibrinogen inhibits sonic hedgehog signaling and impairs neonatal cerebellar development after blood–brain barrier disruption
Source: Proc Natl Acad Sci U S A. 2024 Jul 23;121(31):e2323050121. doi: 10.1073/pnas.2323050121 (PMC11295022; doi:10.1073/pnas.2323050121)
Supplement: Supplementary file 1 — Appendix 01 (PDF) [file pnas.2323050121.sapp.pdf]

PNAS

Supporting Information for

Fibrinogen inhibits sonic hedgehog signaling and impairs neonatal cerebellar development after blood-brain barrier disruption.

Olivia Weaver[a,b,c], Dawn Gano[a,d], Yungui Zhou[b,c], Hosung Kim[e], Reshmi Tognatta[b,c], Zhaoqi Yan[b,c], Jae Kyu Ryu[b,c,d], Caroline Brandt[a,b,c], Trisha Basu[a,b,c], Martin Grana[a], Belinda Cabriga[b,c], Maria del Pilar S. Alzamora[b,c], A. James Barkovich[a,d,f], Katerina Akassoglou[b,c,d], Mark A. Petersen[a,b,c]*

Affiliations:

[a]Department of Pediatrics, University of California San Francisco, San Francisco, CA 94158

[b]Gladstone Institute of Neurological Disease, Gladstone Institutes, San Francisco, CA 94158

[c]Center for Neurovascular Brain Immunology at Gladstone Institutes and University of California San Francisco, San Francisco, CA 94158

[d]Department of Neurology, Weill Institute for Neurosciences, University of California San Francisco, San Francisco, CA 94158

[e]Department of Neurology, Stevens Neuroimaging and Informatics Institute, Keck School of Medicine, University of Southern California, Los Angeles, CA 90033

[f]Department of Radiology & Biomedical Imaging, University of California San Francisco; San Francisco, CA 94143

*Corresponding author: Mark A. Petersen
Email: mark.petersen@ucsf.edu

This PDF file includes:

- Supporting text
- Figures S1 to S4
- Tables S1 to S3
- SI References

Supporting Information

Material and Methods

Human subjects and MRI. Premature newborns <32 weeks' gestation at birth admitted to the intensive care nursery at the University of California, San Francisco (UCSF) between August 2011 and August 2015 were prospectively evaluated and included in this analysis. Exclusion criteria consisted of instability for MRI, congenital infection and/or clinical evidence of malformation or a genetic syndrome. Study participants received a 3-Tesla MRI soon after birth and/or at term-equivalent gestational age or prior to hospital discharge with acquisition parameters as previously described (1, 2). The presence and severity of cerebellar hemorrhage (CBH), white matter injury (3), and intraventricular hemorrhage (4) on MRI were contemporaneously evaluated by a pediatric neuroradiologist (AJB) blinded to clinical history. CBH was classified as absent, mild (<3 foci ≤2 mm), moderate (3-9 foci ≤2 mm or any focus ≤3–5 mm), or severe (≥10 foci ≤2 mm or any focus ≥6 mm). Clinical data were collected prospectively by trained clinical research nurses blinded to imaging. Infection was defined as culture positive sepsis; none had meningitis or were treated presumptively for meningitis; Two participants also had necrotizing enterocolitis defined as Bell's stage II or III (5). Cerebellar volume (mm^3) was determined as previously described (2). Three participants, including two with mild CBH and one with severe CBH, were excluded from volumetric analysis due to motion artifact.

Primary CGNP cultures. Mouse CGNPs were isolated as described (6) with the following modifications. Cerebella from P5-7 C57BL/6 male and female pups were digested in papain (Worthington) in DPBS for 30 minutes at 35°C. After papain inactivation with ovomucoid inhibitor (7) and trituration, the cell suspension was passed through a 40 µm nylon cell strainer (Corning Falcon) to eliminate large non-neuronal cells and obtain a single-cell suspension. To further remove glia, the suspension was incubated for 20 minutes on a poly-D-lysine (PDL, 100 µg/ml)-coated dish and repeated with a fresh dish to remove strongly adherent cells, yielding a final cell suspension of >95% granule neurons and progenitors (6). Cells were seeded onto PDL (500 µg/ml)-coated culture plates and maintained in a 5% CO2, 37°C incubator. Cell culture media was Neurobasal-A (Thermo Fisher Scientific), 1x B27 (Thermo Fisher Scientific), 2 mM GlutaMAX (Thermo Fisher Scientific), 20 mM KCl, and 1% penicillin-streptomycin (Thermo Fisher Scientific). Cells were treated with SHH (#464-SH, R&D Systems) alone or in the presence of fibrinogen (#341578, Millipore-Sigma) at concentrations indicated in the figure legends for up to 3 days in culture. For fibrin-coating experiments, PDL-coated tissue culture plates were incubated with a mixture of fibrinogen (1 µg/mL, #341578, Millipore-Sigma), thrombin (1 U/mL, Millipore-Sigma), and $CaCl_2$ (8.7 mM, Millipore-Sigma) in 20 mM HEPES at 37ºC for 1 hour to form fibrin which was then dried onto the wells at 37ºC overnight. All conditions were tested in triplicate wells and repeated for N = 2 or 3 biological replicates as indicated in the figure legend.

Immunofluorescence. Mice were transcardially perfused with 4% PFA under deep avertin anesthesia. Tissue was removed, post-fixed overnight in 4% paraformaldehyde, cryoprotected in 30% sucrose/PBS, frozen in Tissue-Tek O.C.T. compound (Sakura), cryosectioned into 12 µm sections, and placed on Tissue Tack microscope slides (Polysciences, Inc). Sections were permeabilized in 0.1-0.2% Triton X-100, blocked with 5% BSA or 5% normal donkey serum, and incubated with primary antibodies overnight at 4ºC and then fluorescent secondary antibodies for 1–2 h at room temperature. Slides were coverslipped with SlowFade Gold antifading agent with DAPI (Thermo Fisher Scientific). Primary mouse CGNPs were fixed with 4% PFA for 15 minutes, blocked and permeabilized in 5% normal donkey serum / 0.3% Triton-X100, and incubated with primary antibody overnight at 4ºC and then fluorescent secondary antibodies for 1 h at room temperature. Cell nuclei were visualized with 4′,6-diamidino-2-phenylindole (DAPI).

Images were acquired with an Axioplan II epifluorescence microscope (Carl Zeiss) equipped with dry Plan-Neofluar objectives (10x 0.3 NA, 20x 0.5 NA, or 40x 0.75 NA), an Axiocam HRc CCD camera, and the Axiovision image analysis software; or the BIOREVO BZ-9000 inverted fluorescence microscope (Keyence) equipped with a Nikon CFI 60 Series infinite optical system and Keyence imaging software; All images were processed and analyzed in ImageJ. For animal studies, quantification on two or more nonadjacent sections per mouse was performed on either

thresholded binary images or counting of cells by researchers blind to the treatment group. For in vitro studies, quantification of cells on 5-10 10x images per well was performed using ImageJ.

Immunoblots. Cells or tissue were lysed in RIPA buffer (Thermo Fisher Scientific) supplemented with protease/phosphatase inhibitors (Millipore Sigma) and lysates cleared by centrifuging at 13,000xg for 15 minutes at 4ºC. Equal amounts of protein were loaded in 4%–12% bis-tris gels (Thermo Fisher Scientific) and analyzed by western blotting. Bands were visualized with HRP-conjugated secondary antibodies (Cell Signaling Technology). Densitometry was performed using ImageJ with values for each band normalized to GAPDH loading controls from the same membrane.

Figure S1

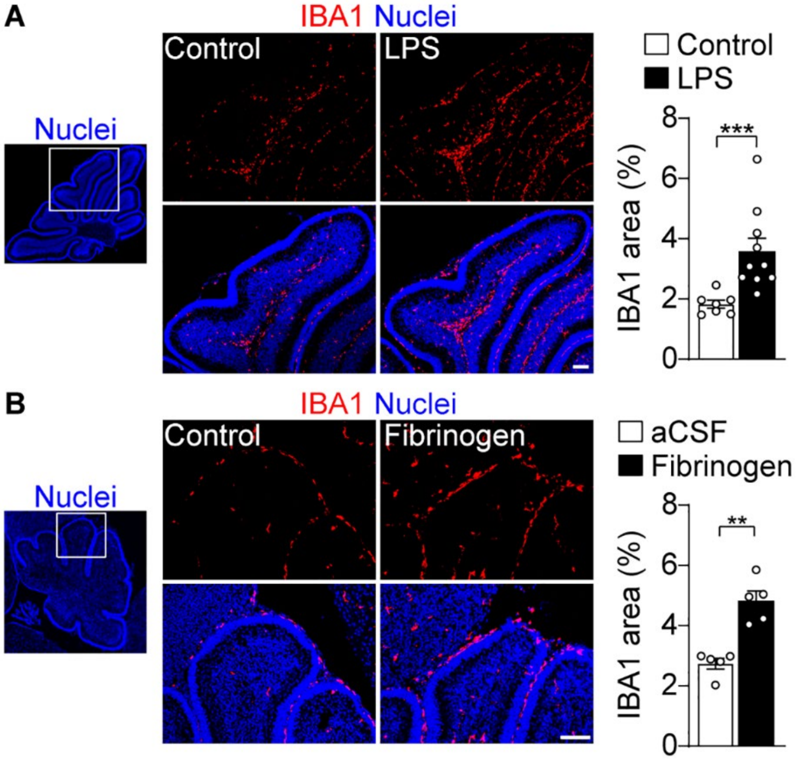

Figure S1. Systemic neonatal inflammation and IC fibrinogen injection increase IBA1⁺ cells in the cerebellum. *(A)* IBA1⁺ (red) cells in the P10 cerebellum, nuclei (blue) labeled with DAPI. Scale bar, 100 µm. Data are mean±s.e.m. from n=7-10 mice per group. ***$p<0.001$, Mann-Whitney. *(B)* IBA1⁺ (red) cells in the P3 cerebellum, nuclei (blue) labeled with DAPI. aCSF or Fibrinogen (2 µl, 5 mg/ml) was injected at P2. Scale bar, 100 µm. Data are mean±s.e.m. from n=5 mice per group. **$p<0.01$, Mann-Whitney.

Figure S2

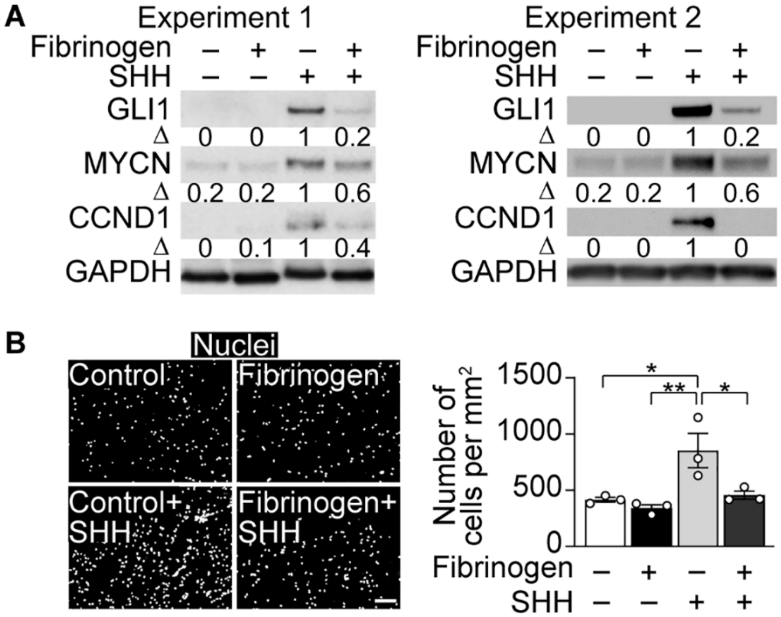

Figure S2. Fibrinogen inhibits the SHH pathway and proliferation of CGNPs. *(A)*GLI1, MYCN, and CCND1 protein levels 24 hours after control or SHH (3 µg/ml) treatment of primary CGNPs in the presence of fibrinogen (2.5 mg/ml) from two independent experiments. Δ, densitometry fold change values. *(B)* Nuclei (white, DAPI) 3 days after control or SHH (3 µg/ml) treatment of primary CGNPs in the presence of fibrinogen (2.5 mg/ml). Scale bar, 100 µm. Data are mean±s.e.m. from n=3 independent experiments. *p<0.05, **p<0.01, two-way ANOVA with Tukey.

Figure S3

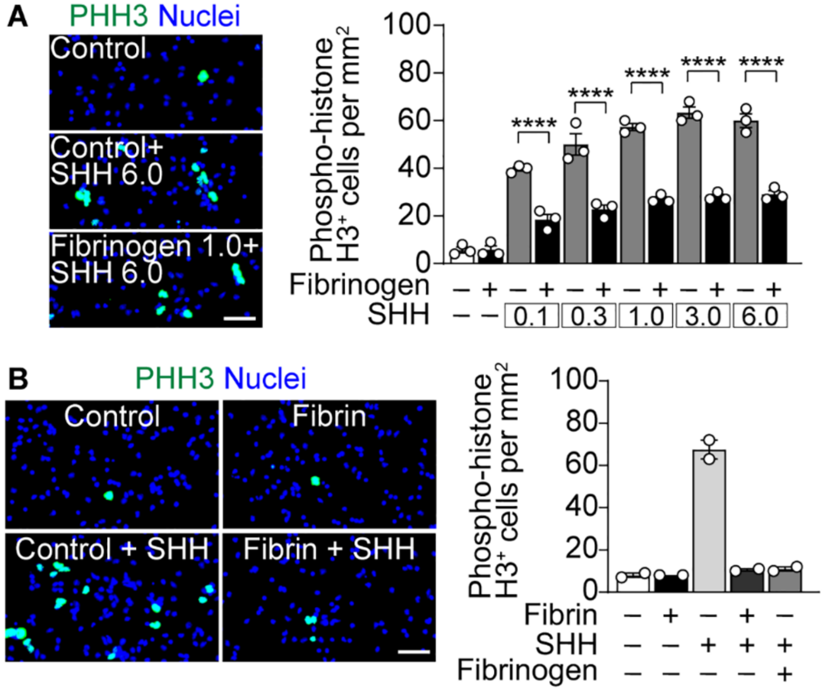

Figure S3. Fibrinogen inhibits proliferation of CGNPs. *(A)* Phospho-histone H3 (PHH3, green) and nuclei (blue, DAPI) 48 hours after control or SHH (0.1, 0.3, 1.0, 3.0, 6.0 µg/ml) treatment of primary CGNPs in the presence of a half-maximal dose of fibrinogen (1.0 mg/ml). Scale bar, 50 µm. Data are mean±s.e.m. from n=3 independent experiments. ****$p<0.0001$, two-way ANOVA with Tukey. *(B)* Phospho-histone H3 (PHH3, green) and nuclei (blue, DAPI) 48 hours after control or SHH (3.0 µg/ml) treatment of primary CGNPs plated on control or fibrin (1 µg/ml)-coated wells. Fibrinogen (2.5 mg/ml) served as a positive control. Scale bar, 50 µm. Data are mean±s.e.m. from n=2 independent experiments.

Figure S4

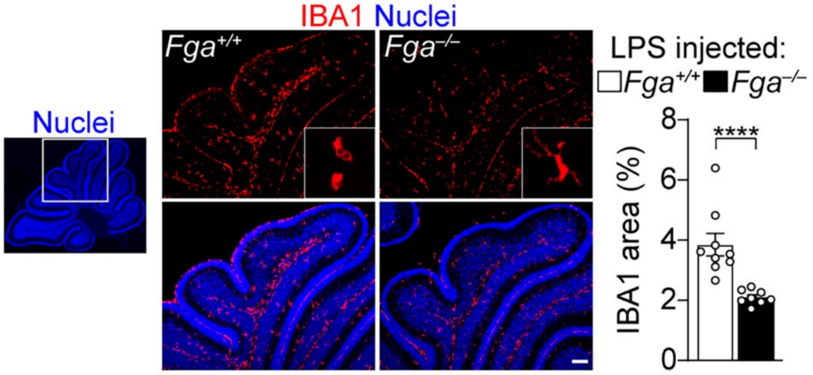

Figure S4. Fibrinogen knockout reduces cerebellar IBA1^{+} cells after systemic neonatal inflammation. IBA1^{+} (red) cells in the P10 cerebellum, nuclei (blue) labeled with DAPI. Scale bar, 100 µm. Inset images, IBA1^{+} cells at higher magnification. *Fga*$^{+/+}$, LPS-treated littermate controls. *Fga*$^{-/-}$, LPS-treated fibrinogen knockout mice. Data are mean±s.e.m. from n=8-9 mice per group. ****p<0.0001, Mann-Whitney.

Table S1: Baseline clinical and imaging characteristics of preterm infants by CBH

	**CBH N=22**	**No CBH N=37**	**P**
**Clinical**			
Gestational age, weeks	27.71 (25.71, 29.43)	29.28 (27.57, 30.56)	0.024
Birthweight, grams	975 (830, 1175)	1200 (1050, 1450)	0.0038
Male, n	8	21	0.18
Prenatal steroids, n	15	30	0.35
Chorioamnionitis, n	4	5	0.72
Sepsis, n	12	11	0.097
NEC, n*	0	2	0.52
Postnatal steroids, n	5	1	0.023
**Imaging**			
CGA last MRI, weeks	35.36 (33.28, 36.57)	35.28 (34.56, 35.57)	0.69
WMI			0.81
Absent	17	28	
Mild	3	5	
Moderate/Severe	2	4	
IVH			0.21
Absent	14	31	
Mild	4	3	
Moderate/Severe	4	3	

*Both infants with NEC also had sepsis

CBH, cerebellar hemorrhage NEC, necrotizing enterocolitis. CGA, corrected gestational age. WMI, white matter injury. IVH, intraventricular hemorrhage.

Table S2: Multivariate regression to assess associations with preterm infant cerebellar volume

	Unadjusted			Adjusted[a]		
	**Beta CbVol**	**95% CI**	**P**	**Beta CbVol**	**95% CI**	**P**
**CBH**						
**Absent**	Ref			Ref		
**Mild**	-687.46	-4430.25, 3055.32	0.71	-201.69	-1555.46, 1152.09	0.77
**Mod/severe**	-2105.81	-4885.36, 673.73	0.14	-1036.44	-2077.89, 5.0	0.051
**Sepsis**	-1359.9	-3783.8, 1064.04	0.27	-134.73	-1002.32, 732.86	0.76
**Postnatal steroids**	-1915.97	-5837.57, 2005.625	0.33	-1615.88	-3111.98, -119.77	0.035
**Gestational age**	471.27	-146.47, 1089.01	0.13	-272.78	-623.94, 78.38	0.13
**Postnatal age at MRI**	475.49	89.83, 861.16	0.017	100.24	-107.62, 308.11	0.34
**Total cerebral volume**	0.069	0.059, 0.073	<0.0001	0.066	0.058, 0.074	<0.0001

[a] Adjusted for CBH severity, sepsis, postnatal steroids, gestational age at birth, postnatal age at MRI, and total cerebral volume.
CbVol, cerebellar volume. CBH, cerebellar hemorrhage.

Table S3: Mean body weight of experimental mouse groups randomized at postnatal day 2 (P2)

**Systemic neonatal inflammation**		
**Experimental group**	**Mean P2 body weight ± s.e.m (g)**	**P, statistical test**
C57Bl/6 pups + saline	1.48 ± 0.03	0.17, Mann Whitney
C57Bl/6 pups + LPS	1.53 ± 0.02	
Fga$^{+/+}$ pups + LPS	1.67 ± 0.10	0.50, Mann-Whitney
Fga$^{-/-}$ pups + LPS	1.63 ± 0.07	
**Intracisternal injections in C57Bl/6 pups**		
**Experimental group**	**Mean P2 body weight ± s.e.m (g)**	**P, statistical test**
aCSF	1.55 ± 0.03	0.99, Mann-Whitney
Fibrinogen	1.55 ± 0.03	
aCSF	1.62 ± 0.03	0.91, one-way ANOVA with Tukey
Fga$^{+/+}$ plasma	1.60 ± 0.03	
Fga$^{-/-}$ plasma	1.60 ± 0.03	

SI References

1. D. Gano *et al.*, Antenatal Exposure to Magnesium Sulfate Is Associated with Reduced Cerebellar Hemorrhage in Preterm Newborns. *J. Pediatr.* **178**, 68-74 (2016).
2. H. Kim *et al.*, Hindbrain regional growth in preterm newborns and its impairment in relation to brain injury. *Hum. Brain Mapp.* **37**, 678-688 (2016).
3. S. P. Miller *et al.*, Comparing the diagnosis of white matter injury in premature newborns with serial MR imaging and transfontanel ultrasonography findings. *AJNR Am. J. Neuroradiol.* **24**, 1661-1669 (2003).
4. L. A. Papile, J. Burstein, R. Burstein, H. Koffler, Incidence and evolution of subependymal and intraventricular hemorrhage: a study of infants with birth weights less than 1,500 gm. *J. Pediatr.* **92**, 529-534 (1978).
5. M. J. Bell *et al.*, Neonatal necrotizing enterocolitis. Therapeutic decisions based upon clinical staging. *Ann. Surg.* **187**, 1-7 (1978).
6. H. Y. Lee, L. A. Greene, C. A. Mason, M. C. Manzini, Isolation and culture of post-natal mouse cerebellar granule neuron progenitor cells and neurons. *J Vis Exp* 10.3791/990 (2009).
7. M. A. Petersen *et al.*, Fibrinogen activates BMP signaling in oligodendrocyte progenitor cells and inhibits remyelination after vascular damage. *Neuron* **96**, 1003-1012 e1007 (2017).
